# Supplementary material for: Causal Relationship between Aging and Anorexia Nervosa: A White-Matter-Microstructure-Mediated Mendelian Randomization Analysis
Source: Biomedicines. 2024 Aug 16;12(8):1874. doi: 10.3390/biomedicines12081874 (PMC11351342; doi:10.3390/biomedicines12081874)
Supplement: Supplementary file 1 [file biomedicines-12-01874-s001.zip › Supplementary Figure/Figure S8/Leaveoneout plot of MR analysis for TL to ubm-b-2018.pdf]

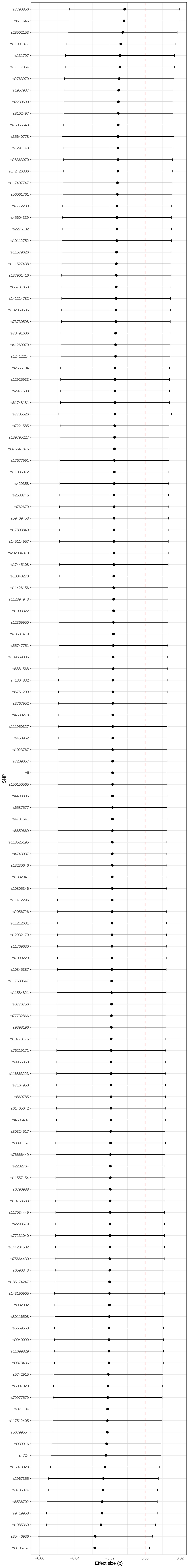

rs7790856
rs611646
rs28502153
rs11991877
rs131797
rs11117354
rs2763979
rs1957937
rs2230590
rs8102497
rs76065543
rs35640778
rs1291143
rs28363070
rs142426306
rs117407747
rs56061761
rs7772289
rs45604339
rs2276182
rs10112752
rs11579626
rs111527438
rs137901416
rs66731853
rs141214782
rs182059586
rs73730598
rs78491606
rs41269079
rs12412214
rs2555104
rs12925933
rs2977608
rs61748181
rs7705526
rs7221585
rs139795227
rs376641875
rs17677991
rs11085072
rs429358
rs2538745
rs762679
rs59409453
rs17803849
rs145114957
rs202034370
rs17445108
rs10840270
rs11426156
rs112394943
rs1003322
rs12369950
rs73581419
rs55747751
rs139669835
rs6881568
rs41304832
rs6751209
rs3767952
rs4530278
rs111950327
rs450962
rs1023767
rs7209057
All
rs150150565
rs4498805
rs6587577
rs4731541
rs6659669
rs113525195
rs4743037
rs13230646
rs1332941
rs10805346
rs11412296
rs2056726
rs11212631
rs12932179
rs11769630
rs7099229
rs10845387
rs117630647
rs11584821
rs6776756
rs77732866
rs9398196
rs10773176
rs76219171
rs9955360
rs116863223
rs7164950
rs869785
rs61405042
rs4695407
rs80324517
rs3891167
rs76666449
rs2282764
rs11557154
rs6790988
rs10768683
rs117034449
rs2293579
rs77231040
rs144204502
rs75664430
rs6590343
rs185174247
rs143190905
rs932002
rs80116508
rs6669563
rs9940099
rs11699829
rs9878436
rs5742915
rs6007020
rs79977579
rs871134
rs117512405
rs56799554
rs939916
rs4724
rs16978028
rs2967355
rs3785074
rs6536702
rs9419958
rs1985369
rs35446936
rs8105767
SNP
-0.06
-0.04
-0.02
0.00
0.02
Effect size (b)
